# Biomarkers for assessing pain and pain relief in the neonatal intensive care unit

**DOI:** 10.3389/fpain.2024.1343551

**Published:** 2024-02-15

**Authors:** Judith A. ten Barge, Mathilde Baudat, Naomi J. Meesters, Alida Kindt, Elbert A. Joosten, Irwin K.M. Reiss, Sinno H.P. Simons, Gerbrich E. van den Bosch

**Affiliations:** ^1^ Department of Neonatal and Pediatric Intensive Care, Division of Neonatology, Erasmus MC—Sophia Children’s Hospital, Rotterdam, Netherlands; ^2^ Department of Anesthesiology and Pain Management, Maastricht University Medical Centre+, Maastricht, Netherlands; ^3^ Department of Translational Neuroscience, School of Mental Health and Neuroscience, Maastricht University, Maastricht, Netherlands; ^4^ Metabolomics and Analytics Center, Leiden Academic Centre for Drug Research, Leiden University, Leiden, Netherlands

**Keywords:** analgesic therapy, biomarkers, infant, neonatal intensive care, pain, pain measurement

## Abstract

Newborns admitted to the neonatal intensive care unit (NICU) regularly undergo painful procedures and may face various painful conditions such as postoperative pain. Optimal management of pain in these vulnerable preterm and term born neonates is crucial to ensure their comfort and prevent negative consequences of neonatal pain. This entails accurate and timely identification of pain, non-pharmacological pain treatment and if needed administration of analgesic therapy, evaluation of treatment effectiveness, and monitoring of adverse effects. Despite the widely recognized importance of pain management, pain assessment in neonates has thus far proven to be a challenge. As self-report, the gold standard for pain assessment, is not possible in neonates, other methods are needed. Several observational pain scales have been developed, but these often rely on snapshot and largely subjective observations and may fail to capture pain in certain conditions. Incorporation of biomarkers alongside observational pain scores holds promise in enhancing pain assessment and, by extension, optimizing pain treatment and neonatal outcomes. This review explores the possibilities of integrating biomarkers in pain assessment in the NICU.

## 1 Introduction

Newborns admitted to the neonatal intensive care unit (NICU) are frequently exposed to painful procedures and conditions, necessitating adequate pain treatment with non-pharmacological interventions and, if needed, analgesic therapy with for instance paracetamol and opioids (1). Providing optimal pain treatment to preterm born and critically ill neonates is crucial, since exposure to pain during this vulnerable period is associated with harmful short- and long-term effects, including increased complications and impaired neurodevelopment (2–6).

For effective pain treatment, it is necessary to regularly assess the neonate's pain level. Since neonates are unable to verbally communicate their pain level, pain assessment in the NICU largely relies on clinical observations including the use of observational pain scales. Several pain scales are available for use in neonates, both for preterm and term born neonates and for acute and prolonged pain assessment (7). These scales depend on behavioral indicators of pain, such as crying and facial expression. However, these scores are all mainly subjective, snapshot assessments and in certain conditions the validity of these scales may be questioned. For instance, preterm neonates suffering from the severe and very painful gastrointestinal condition necrotizing enterocolitis often exhibit few movements and a blank facial expression due to their critical illness, which may result in a low pain score despite presence of severe pain (8, 9). Moreover, behavioral pain scores require observation of the neonate by a trained NICU professional, and are thus time-consuming and dependent on staff-availability. These scores are not available continuously, whereas neonates’ pain behaviors may vary over time, especially in critically ill and extremely low birth weight neonates lacking the energy reserves required to exhibit these pain-related behaviors.

Biomarkers are a useful addition to the NICU professional's pain assessment toolbox. A biomarker is “a defined characteristic that is measured as an indicator of normal biological processes, pathogenic processes or responses to an exposure or intervention” (10). This entails molecular, histologic, physiologic and radiographic characteristics. Based on their application, seven subtypes of biomarkers have been defined, namely diagnostic-, monitoring-, response-, predictive-, prognostic-, safety-, and susceptibility/risk-biomarkers (11). One biomarker may fit multiple purposes and then thus belongs to multiple subtypes. In general, biomarkers don’t play a major role in assessing pain and pain relief in the NICU, except physiological parameters (e.g., heart rate) that are incorporated in some observational pain scales as well (7). For optimal pain management, it is essential to quickly detect pain, initiate appropriate treatment, and assess the treatment’s effectiveness and safety. Biomarkers have the potential to improve each of these stages: monitoring-biomarkers contribute to the detection of pain, response-biomarkers advance the evaluation of pain treatment, and safety-biomarkers allow the (early) detection of adverse effects. This review provides an overview of biomarkers that might contribute to optimizing pain management in the NICU, and consequently, improve neonatal outcomes. The available evidence in neonates is reviewed, and promising biomarkers identified in preclinical studies are discussed.

## 2 The ideal biomarker for assessing pain and pain relief

The usefulness of a biomarker for assessing pain and pain relief in the NICU is determined by several properties (12). First and foremost, the biomarker needs to be valid, demonstrating a strong association with pain supported by sufficient evidence. A high sensitivity and specificity for pain are needed to prevent unneeded, insufficient or excessive administration of analgesia. Moreover, the accessibility of the biomarker is crucial, encompassing both the ease of biomarker collection and timely availability of the measurement result. A biomarker for assessing pain in the NICU should be collected non-invasively without causing additional pain. Furthermore, the ideal monitoring biomarker for pain assessment in the NICU should respond rapidly to pain and should be measured quickly (preferably continuously), enabling prompt detection and intervention to minimize pain exposure. Additionally, for a response biomarker, it is essential that a change in the biomarker after administration of pain treatment reflects the analgesic's anti-nociceptive effect, rather than an adverse effect. An important quality for a safety biomarker is that it can be measured before any serious adverse effects of analgesics have occurred, to enable timely intervention.

This review incorporates two methods for assessing the validity of potential biomarkers for pain. The first is to examine the agreement between biomarker levels and the “gold standard” for pain assessment, defined as criterion validity in the COSMIN guidelines (13). Observational pain scores are currently considered the closest approximation to a gold standard for pain measurement in neonates. Consequently, many studies examine the validity of potential pain biomarkers by correlating biomarker levels with observational pain scores. The second method is to determine the construct validity by testing the hypothesis that administering pain relief reduces the change in biomarker levels observed in controls.

## 3 Monitoring- and response-biomarkers

**Figure 1:**
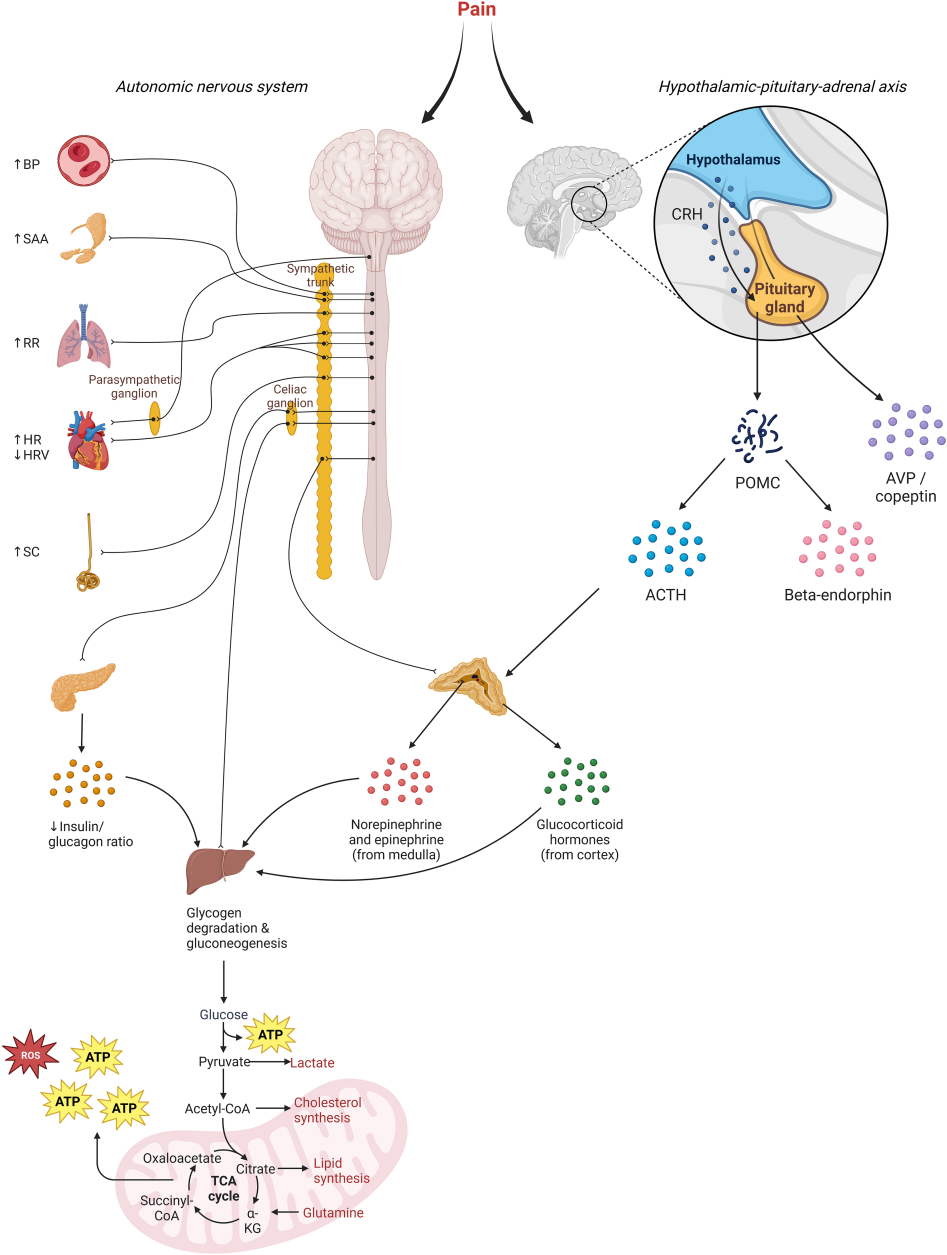
Illustration of the underlying mechanisms for pain biomarkers. Increased sympathetic activity results in rises in blood pressure (BP), salivary amylase (SAA), respiratory rate (RR), heart rate (HR), and skin conduction (SC). Decreased parasympathetic activity diminishes heart rate variability. Additionally, heightened sympathetic activity triggers increased secretion of catecholamines and glucagon from the adrenal gland and pancreas. Release of corticotroponin-releasing hormone (CRH) by the hypothalamus stimulates the pituitary to synthesize precursor protein proopiomelanocortin (POMC), which is subsequently cleaved into adrenocorticotropin (ACTH) and beta-endorphin, among others. ACTH causes secretion of glucocorticoid hormones. Overall, this hormonal response causes a catabolic response aimed at metabolizing energy storages, which releases reactive-oxygen species (ROS).

As illustrated in Figure 1, pain activates the nociceptive system and evokes a stress response involving the sympathetic nervous system and hypothalamic-pituitary-adrenal (HPA) axis (14). This results in cerebral, autonomic, and hormonal responses, along with changes in different biochemical processes. These biomarkers might be used to detect pain (monitoring-biomarkers) and/or evaluate the effectiveness of pain treatment including analgesic therapy (response-biomarkers). The observed stress response is not specific to pain; other stressors may activate the sympathetic nervous system and HPA-axis as well.

Since the cerebral cortex is involved in the perception of pain (15), imaging neurophysiological responses may provide the closest estimate of pain perception, aside from verbal reports. Several studies have identified neurophysiological responses in neonates undergoing painful procedures and some trials assessing analgesic efficacy have used noxious-evoked brain activity as study endpoint (16). Noxious-evoked brain activity has been recorded in neonates using near-infrared spectroscopy (NIRS), electroencephalography (EEG), functional magnetic resonance imaging (fMRI), and electromyography (EMG). A disadvantage of NIRS is that this technique relies on the assumption that changes in oxygenation directly correspond with changes in brain activity, whilst this association may be influenced by other factors (17). A limitation of EEG is its relatively poor spatial resolution and a limitation of EMG its relatively low specificity. The utility of fMRI for pain assessment in the NICU is limited by logistic challenges. For further information about neurophysiological responses to pain, the reader is referred to this review (16).

### 3.1 Autonomic

The autonomic nervous system regulates internal physical functions and maintains homeostasis through its two divisions: the sympathetic and parasympathetic nervous system, which exert opposite effects that can be summarized as “fight or flight” and “rest and digest”, respectively (18). Due to its survival importance, pain stimulates the sympathetic nervous system (19), leading to increased plasma catecholamine levels and changes in for instance blood pressure and heart rate, the latter being continuously monitored in neonates admitted to the NICU.

#### 3.1.1 Heart rate

Painful procedures in neonates have been shown to increase heart rate (20–29), mediated by sympathetic stimulation of β1 receptors in the heart (18). The rise in heart rate following a painful procedure occurs within seconds. Studies assessing the agreement between observational pain scores and heart rate changes during painful procedures in neonates have reported conflicting results, with two studies reporting a moderate correlation and another study no correlation (20, 26, 27). This discrepancy may be attributed to maturational differences, since the former two studies included preterm born neonates whereas the latter study included full-term neonates. However, Van der Vaart et al. found that heart rate responses to pain increase with postmenstrual age (30). Due to the immaturity of their brainstem, pain in preterm neonates may also cause apnea of prematurity, which is defined as a cessation of breathing for over 15–20 s combined with oxygen saturation and/or bradycardia (31). In addition to maturational aspects, the heart rate response to acute pain may be affected by sex, with male neonates displaying a larger increase in heart rate (29). The specificity of heart rate for assessment of neonatal pain is likely limited by the fact that factors such as body temperature (e.g., fever, therapeutic hypothermia) also affect heart rate (32).

For assessing prolonged pain, heart rate seems to have only limited value. Studies have found that during the postoperative period, neonates’ heart rate did not correlate well with prolonged pain scores. Mean heart rate did not differ significantly between postoperative neonates with low and high prolonged pain (EDIN) scores (33). Similarly, heart rate at three, six, and nine hours postoperatively did not correlate well with pain scores at these time points (34). Correlations between heart rate and pain scores during the postoperative period varied considerably between individuals and were higher in those with higher levels of pain (35). The limited correlation between heart rate and prolonged pain scores may be due to adaptation of autonomic responses to prolonged pain and/or the influence of other factors (e.g., diseases or medication) on heart rate. Currently available continuous heart rate data provide new opportunities for more detailed analyses of its relationship with neonatal pain and comfort, as these data enable investigation of heart rate patterns over time rather than means or measurements at fixed time points.

Changes in heart rate may be used in bedside dashboards to evaluate the effectiveness of started or intensified analgesic therapy, as opioids have been shown to reduce heart rate in two RCTs, one among mechanically ventilated neonates and one in neonates undergoing endotracheal suctioning (36, 37). However, neither of these RCTs investigated the correlation between heart rate responses and pain scores, thereby hampering the distinction between the effects of pain relief and possible direct effects of opioids on heart rate.

#### 3.1.2 Heart rate variability

Short-term variations in heart rate primarily reflect changing levels of parasympathetic and sympathetic stimulation of the sinoatrial node. Using spectral analysis, variations in heart rate can be quantified as a function of their frequency. Fluctuations in heart rate at high frequencies (>0.15 Hz) are due to changes in parasympathetic activity, whereas at low frequencies (<0.15 Hz), these changes can be attributed to changes in either sympathetic or parasympathetic activity (38). Parasympathetic stimulation of the heart is rhythmically connected to respiratory activity, with parasympathetic activity decreasing during inspiration and rising during expiration, resulting in transient increases and decreases in heart rate, respectively.

The Newborn Infant Parasympathetic Evaluation (NIPE) Index is a measure of heart rate variability at high frequencies, reflecting parasympathetic activity (39). Decreases in NIPE index indicate pain, whilst increases indicate improved comfort. Several studies have found that the (instant) NIPE index decreases during painful procedures and is inversely correlated with pain scores in neonates (40–44). However, other studies found no correlation with pain scores (45, 46). Moreover, the reported diagnostic performance varied considerably between studies, with the AUC ranging from 0.56 to 0.93 (41, 42, 44, 46–48). NIPE generally performed better in neonates with higher levels of pain compared with those with mild pain.

The usefulness of NIPE for prolonged pain assessment has been studied less, but associations with observational scores for prolonged pain have been found in neonates undergoing mechanical ventilation and in the postoperative period (33, 49–51).

Decreases in NIPE value can be detected in neonates under anesthesia and may be indicative of insufficient antinociception, as NIPE values have been shown to rise after administration of opioids in neonates with NIPE values below 50 (indicating pain) but remain unchanged in those with NIPE values above 50 prior to opioid administration (52). Similarly, EMLA cream mitigated the reduction in heart rate variability observed during venipuncture (53). During the postoperative period, pain treatment with morphine has been associated with an increase in heart rate variability at high frequencies, although this was no longer significant after adjusting for confounders (54). The latter two studies used a different method than NIPE for spectral analysis of heart rate variability.

An advantage of NIPE for use in the NICU setting is that it continuously assesses pain in a non-invasive manner. Limitations include its inapplicability in neonates with a postconceptional age below 26 weeks and the need for an additional device, thereby limiting its usefulness in low-resource settings.

#### 3.1.3 Blood pressure

Increases in neonates’ blood pressure have been detected during painful procedures and these increases are greater during more invasive procedures (22, 28). Validity of blood pressure for assessment of prolonged pain in neonates is limited (34). The correlation between blood pressure and behavioral scores for prolonged pain has been shown to vary considerably between subjects, with higher correlations in older infants compared with neonates and reduced correlations in the presence of systemic inflammatory response syndrome (SIRS)/sepsis (35). Moreover, higher correlations between blood pressure and prolonged pain scores were observed in those with higher levels of pain (35). Similarly, the correlation between prolonged pain scores and blood pressure variability was increased in those with higher pain levels (35).

Pain treatment may dampen blood pressure responses to pain in neonates. However, treatment with opioids can also reduce blood pressure by inducing peripheral arteriolar and venous dilation, and may cause hypotension if dosed excessively (55–59). In an RCT among mechanically ventilated neonates, no significant effect of morphine treatment on blood pressure variability was identified (55).

The utility of blood pressure (variability) for pain assessment in the NICU is limited by the fact that blood pressure is affected by various clinical factors and that blood pressure is only measured continuously in the subgroup of patients with an in-dwelling arterial catheter. Placing a peripheral in-dwelling arterial catheter is painful and is mostly performed on indication (e.g., cardiovascular instability or frequent blood sampling). Non-invasive blood pressure (NIPB) monitoring with a blood pressure cuff provides intermittent measurements and is uncomfortable as well. Recent advances in non-invasive blood pressure measurement techniques may enable cuffless, continuous blood pressure measurement in the future (60).

#### 3.1.4 Skin conductance

Sympathetic stimulation induces sweating from the palmar and plantar sweat glands (18), leading to a decrease in skin resistance and consequent increase in skin conductance, until the sweat is reabsorbed and skin resistance increases, causing skin conductance to decline (61). This change in skin conductance can be measured with a skin conductance algesimeter. Each burst fired by the skin sympathetic nerve causes a peak in skin conductance, resulting in a wavelike pattern. Three characteristics can be derived from this pattern, namely number of waves per second, amplitude (i.e., peak size), and basal level.

Painful procedures in neonates have been shown to cause an increase in the number of peaks per second (27, 62–67). This increase was greater than that observed during non-painful tactile stimulation, correlated with observational pain scores (27, 62–64, 67), and correlated inversely with the NIPE index (43). Moreover, an increase in peak size has been detected in response to pain, but not tactile stimulation (62).

Skin conductance responses to painful procedures can be observed in neonates receiving analgesia or sedation (65), but are obliterated by neuromuscular blockade (68). Moreover, skin conductance responses (increased number of peaks per second) have been observed during persistent pain or stress, as experienced postoperatively and during mechanical ventilation (69–72), and a cutoff value of 0.1 fluctuations of skin conductance per second enabled discrimination between pain requiring urgent attention and well-controlled pain in postoperative children (69–71).

Concerns have been raised regarding the specificity of skin conductance measures for pain, since skin temperature is also highly correlated with skin conductance (73), reflecting the role of sympathetic stimulation in autoregulation of skin temperature. Furthermore, significant variation in skin conductance responses is present between patients, as well as within patients (63, 68). A study in extremely preterm neonates found no significant changes in the number of peaks per second during painful procedures, possibly due to their prematurity or the administration of sucrose (21). A recent scoping review on the validity of skin conductance for assessing acute pain in infants identified inconsistent results (74). Nevertheless, two studies evaluating the performance of skin conductance found an acceptable discrimination for pain overall and an excellent discrimination for moderate to severe pain (62, 64).

An advantage of skin conductance for use in the NICU setting is that it enables continuous evaluation of pain levels. A disadvantage is that it requires attaching an extra sensor to the skin. Extremely preterm neonates have very fragile skin and may experience medical-adhesive related skin injury (75). Estimates of the validity of skin conductance for pain assessment in neonates are variable and generally moderate, although higher for more severe pain.

#### 3.1.5 Alpha-amylase

Salivary alpha amylase (sAA) is one of the main enzymes present in saliva and it contributes to the digestion of starch. As sympathetic stimulation of the salivary gland affects saliva secretion, sAA may be used as a marker of sympathetic activity (76). However, the usefulness of sAA as a marker for pain remains controversial. One study in neonates undergoing painful procedures detected no significant changes in sAA levels and found a high inter- and intra-subject variability (77). A study evaluating sAA responses to inoculation in neonates at different ages found stress-related sAA increases at 6 and 12 months, but not at 2 and 24 months of age (78). The lack of a detectable sAA response at 2 months may be explained by the fact that sympathetic innervation of the salivary glands develops postnatally, making sAA a marker with limited validity for the preterm/NICU population (79). Moreover, collecting sufficient saliva for sAA measurement may be challenging in preterm neonates.

#### 3.1.6 Other autonomic biomarkers

In addition to the above-mentioned autonomic biomarkers, respiratory rate and oxygen saturation may also be affected by acute pain. During painful procedures in neonates, respiratory rate has been shown to increase, reflecting sympathetic stimulation (25, 28). Oxygen saturation, on the other hand, has been shown to decrease during painful procedures and this decrease is larger in the most preterm neonates (20, 21, 25, 28, 80). Pain relief can reduce this pain-related hypoxemia (81). In case of treatment with opioids, reductions in respiratory rate may also be due to direct respiratory repression by opioids rather than via its analgesic effects (56).

**Figure 2:**
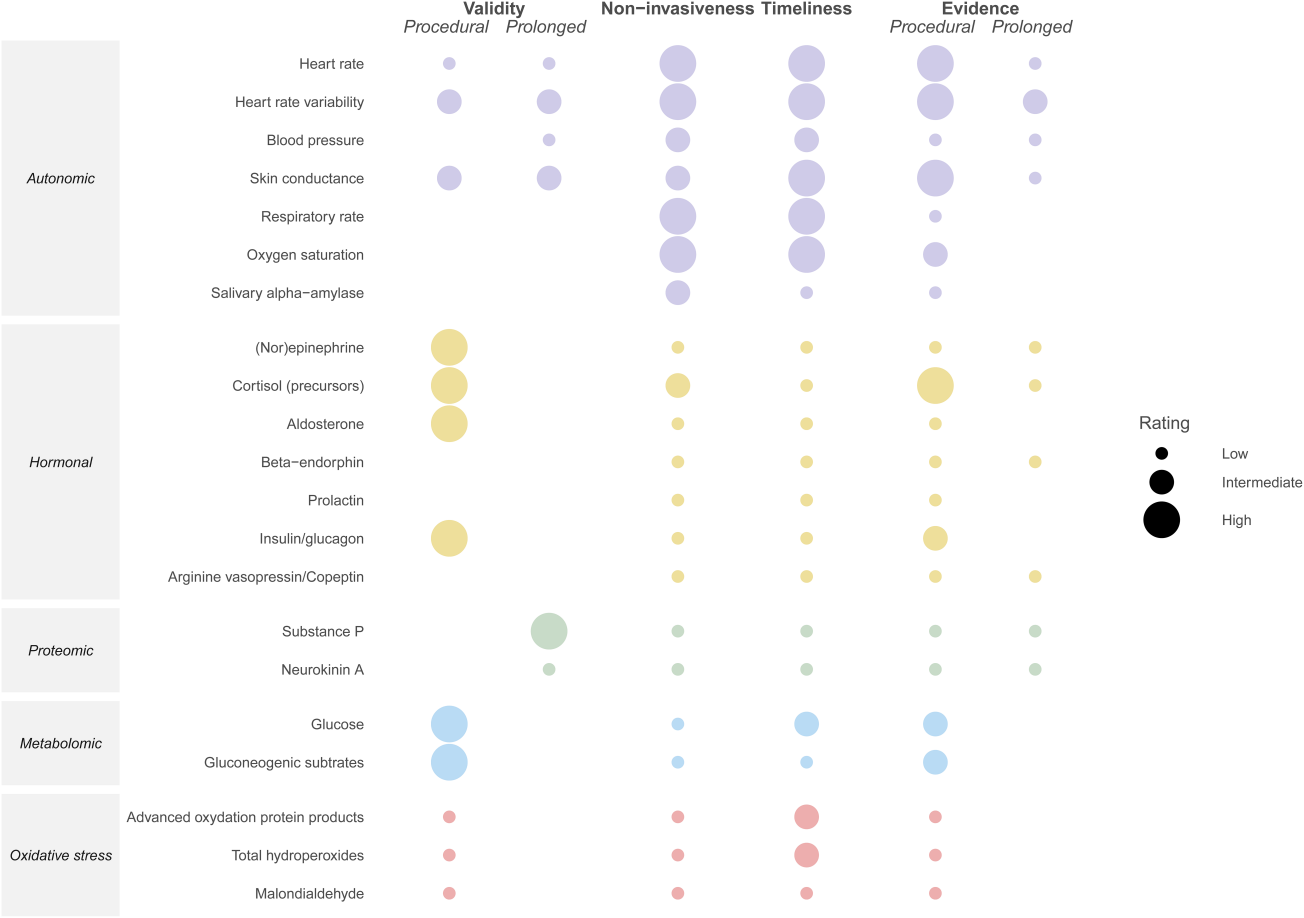
Illustration of the comparative validity, non-invasiveness, timeliness, and amount of available evidence from clinical studies per biomarker, with the validity and amount of available evidence stratified by type of pain (i.e., procedural vs. prolonged). Validity: high = effect of pain relief confirmed in placebo-controlled RCT, average Pearson's correlation ≥0.70 or AUC ≥ 0.80; intermediate = average correlation 0.40–0.70 or AUC 0.70–0.80; low = average correlation <0.40 or AUC < 0.70; no rating = none of these measures available (83). Non-invasiveness: high = no additional patient interventions required for biomarker collection; intermediate = additional but non-painful intervention required (e.g., saliva collection, application of electrode); low = painful procedure required (e.g., venipuncture) (84). Timeliness: high = can be monitored continuously; intermediate = bedside or rapid (<10 min) assay available; low = no rapid assay available. Evidence: high = evaluated in ≥10 studies in neonates; intermediate = evaluated in 5–10 studies in neonates; low = evaluated in 1–5 studies in neonates; no rating = no studies in neonates identified. The ratings for blood pressure apply to the situation in which a blood pressure cuff is used.

Although these physiological data have the benefit of being readily available in the NICU, their utility for pain assessment is limited by the fact that they are also altered by the patient's clinical condition and medical interventions, rendering them less specific (35, 82) (Figure 2).

### 3.2 Hormones

#### 3.2.1 Catecholamines

Sympathetic activity results in the release of catecholamines (i.e., epinephrine and norepinephrine) from the adrenal medulla and the release of norepinephrine from presynaptic nerve terminals, resulting in increased catecholamine levels. In a landmark randomized controlled trial by Anand et al, it was shown that preterm neonates undergoing surgery without analgesia (i.e., only anesthesia) exhibited a greater hormonal response, including higher epinephrine and norepinephrine responses (3). The catecholamine response during surgery in neonates is more extreme than that observed in adults (85), and is positively correlated to the degree of surgical stress (86). Less invasive procedures, such as endotracheal suctioning, have also been shown to mount a significant catecholamine response in preterm neonates (87).

In addition to painful procedures, prolonged pain or stress—as observed during mechanical ventilation—can evoke a catecholamine response proportional to the severity of illness (88). This catecholamine response is attenuated by analgesic therapy with opioids, as has been established in several clinical placebo-controlled RCTs (58, 89, 90). Moreover, opioids have been shown to reduce urinary concentrations of the catecholamine metabolites metanephrine and normetanephrine (91). A preclinical study in rats found that pain increased normetanephrine levels in the dorsal half of the spinal cord, and that normetanephrine levels were further elevated by administration of morphine (92). Lidocaine, on the other hand, suppressed the increase in spinal normetanephrine induced by noxious stimuli. These findings suggest that spinal normetanephrine is not a marker of pain but of (noradrenergic) descending pathways activation (92).

Preclinical studies have shown that plasma levels of catestatin, an epitope of the glycoprotein chromogranin A which is co-released with catecholamines, are diminished following traumatic bone fracture or ovariohysterectomy in dogs (93–95). Plasma levels of catestatin were unaffected by morphine administration and correlated weakly with pain scores (94, 95), so its validity as a marker for pain is yet to be established.

The utility of catecholamines for bedside pain assessment in the NICU is limited by the fact that they are measured in blood and that catecholamine assays are mostly laborious and time-consuming.

#### 3.2.2 Cortisol

In addition to sympathetic stimulation, the stress response to painful stimuli consists of activation of the hypothalamic-pituitary-adrenal (HPA) axis, thereby enabling mobilization of energy from storage sites to the heart, muscles, and brain (96). Activation of neurons in the hypothalamus results in the secretion of corticotrophin releasing hormone (CRH) and arginine vasopressin (AVP), inducing secretion of ACTH by the anterior pituitary, and consequently secretion of cortisol by the adrenal cortex (Figure 1).

Cortisol responses can be measured in blood, saliva, urine and hair (97, 98). The former three provide potential measures of acute stress, whereas hair cortisol concentration offers a way to analyze chronic stress in the previous period in older infants and adults. For assessment of acute pain in neonates, salivary cortisol levels are most used because of its relatively easy accessibility. Measurement of hair cortisol levels is often not feasible in neonates due to too little hair.

A systematic review on salivary cortisol reactivity in infants found that painful stressors evoked the strongest cortisol response, in comparison with mild physical stressors and psychological stressors, and that the cortisol response to pain was especially prominent in younger infants and decreased with postnatal age (99). Salivary cortisol levels in neonates peak 20–25 min after the stressful event (100), which limits their clinical usefulness for pain assessment in neonates admitted to the NICU. Out of three studies that evaluated the use of salivary cortisol measurements for monitoring procedural pain in the NICU, two identified a significant correlation between pain scores during the procedure and peak cortisol levels and the third found no such correlation (27, 101, 102). However, in the latter study, pain was assessed using the CRIES score, which has been developed for assessment of postoperative rather than procedural pain (103). Furthermore, a preclinical rat model revealed that plasma corticosterone, the rodent equivalent to cortisol, is increased after acute neonatal inflammatory pain for up to 7 days post-injury (104). The long lasting effect of a single painful intervention on plasma corticosterone levels suggests that cortisol levels may be affected by previous painful procedures.

Cortisol levels in neonates have been studied relatively little during prolonged pain or stress. In a group of non-invasively ventilated neonates, those receiving a more painful ventilation method exhibited higher cortisol levels (105). In term born neonates undergoing cardiac surgery, plasma cortisol levels were shown to increase during the first 18 h and remain elevated up to 48 h postoperatively, whereas urinary cortisol hardly changed (54). The previously mentioned RCT by Anand et al., which assessed hormonal and metabolic responses in preterm neonates undergoing cardiac surgery with and without fentanyl analgesia, found significantly higher corticosterone and 11-deoxycorticol but not cortisol responses during surgery in the placebo group (3). Similarly, Orsini et al. found that fentanyl significantly reduced 11-deoxycortisol but not cortisol levels in preterm neonates with respiratory distress syndrome (106). Guinsburg et al. found that in mechanically ventilated preterm neonates, fentanyl showed a tendency to reduce cortisol and significantly reduced 11-deoxycortisol levels (36). The finding of significant differences in these precursors steroid hormones but not cortisol itself may be attributed to a relative deficiency of the more distal enzymes of the steroidogenesis pathway in the immature adrenal cortex (107).

A limitation of using salivary cortisol measurements for pain assessment in the NICU is that it may be difficult to collect sufficient saliva from preterm neonates (108). Moreover, cortisol assays are generally time-consuming, rendering them unsuitable for pain assessment in clinical practice (109).

#### 3.2.3 Other hormones

Alterations in various other (stress) hormones have been detected in neonates undergoing surgery, including increases in aldosterone, beta-endorphin and prolactin, and a decrease in the insulin/glucagon molar ratio (3, 85, 110–114). Overall, the hormonal response during surgery results in a catabolic state with several metabolic derangements. The trial by Anand et al. showed that the alterations in aldosterone and insulin/glucagon molar ratio are mitigated by analgesic therapy (3).

Increases in the endogenous opioid beta-endorphin have also been observed in neonates undergoing mechanical ventilation and are reduced by treatment with opioids (115). In an RCT of neonates undergoing endotracheal suctioning, no changes in beta-endorphin levels were observed, neither in the analgesia group nor in the placebo group, which may have been due to not sampling at the right time point (37). Longitudinal evaluation of plasma beta-endorphin levels after painful and non-painful stimuli in adult mice revealed a stimulus-specific pattern of beta-endorphin release, with painful stimuli resulting in a later and more prolonged peak in beta-endorphin levels than non-painful stressors (116). This highlights the importance of timing for sampling plasma beta-endorphin as a marker of pain. Changes in beta-endorphin are not limited to the plasma but have also been observed in the cortex of rats exposed to neonatal inflammatory pain a week post-injury (104). Although less marked, similar increases in other endogenous opioids like met-enkephalin were observed in the midbrain and spinal cord of these rats exposed to neonatal acute inflammatory pain (104).

Moreover, increases in plasma levels of arginine vasopressin (AVP) have been detected in neonates after surgery, despite normal sodium concentrations, osmolality, and cardiovascular parameters, suggesting a role for AVP as a marker of pain and/or stress (117). Similarly, in mechanically ventilated neonates, bursts of arginine vasopressin secretion uncorrelated with plasma osmolality have been observed (118). In neonates undergoing circumcision, on the other hand, no changes in AVP have been detected (119). Copeptin, a surrogate marker of AVP, has also been related to neonatal stress (120).

### 3.3 Proteomics

Proteomics refers to the identification and quantification of the entire set of proteins in a biological specimen. Contrary to the genome, the proteome is dynamic and environmental factors such as pain may alter protein expression. In the context of neonatology, gestational age and postnatal age can significantly affect the proteome, with the expression of many proteins, including drug metabolizing enzymes, changing over time (121–123). Several candidate proteins for pain have been proposed in adults, including neurotrophic factors, neuropeptides, and cytokines (124).

Substance-P (SP) and neurokinin-A (NKA), two peptides likely involved in the transmission and modulation of noxious stimuli, have been proposed as biomarkers of acute pain in neonates. Reference values for plasma SP and NKA levels have been established in neonates (125). Increases in SP and decreases in NKA have been detected after painful procedures in neonates (126, 127). Moreover, increased levels of SP and NKA have been found in neonates with the painful intestinal disease necrotizing enterocolitis, and SP correlated with observational pain scores (128).

An alternative approach to this “candidate protein approach”, which searches for a single protein indicative of pain, is to study the combination of multiple proteins with high throughput analyses and create a so-called “biosignature” (129). Proteomic profiling of skin biopsies 24 h after a skin incision in adult humans identified distinct protein signatures in subjects with a high and subjects with a low hyperalgesia response, with proteins related to anti-inflammatory processes predominating in the low responders and proteins related to a proteolytic environment and persistent inflammation in the high responders (130).

As only very few studies have evaluated proteomics in relation to acute pain and analgesic therapy in humans, additional insights mainly originate from animal studies. In dogs undergoing surgery, significant decreases in plasma levels of complement C-3, complement factor B, complement factor D, transthyretin, and proteins associated with lipid, cholesterol, and glucose metabolism were observed (131). Many of these changes were mitigated by analgesic therapy. Pathway analysis revealed that most of the significantly altered proteins were involved in blood coagulation and the immune response.

In calves undergoing the painful dehorning procedure, a gradual change in the proteome was observed following the procedure, with significant changes after 24 and 96 h (132). Proteins exhibiting significant changes after 24 h were primarily associated with inflammatory and immune responses, whereas those altered after 96 h were linked to stress, pain, and wound healing pathways. Differentially expressed proteins included complement C5a, nerve-growth-factor (NGF), and endopin, among others. Endopin 1 and 2B were decreased at 24 h after intervention and increased 96 h after intervention, respectively. Endopins are co-secreted with the endogenous opioid (met-)enkephalin, which, as described in the previous section, is known to be increased after neonatal inflammatory pain (104, 132, 133).

Disadvantages of using proteomic biomarkers for pain assessment in the NICU include the need to collect blood and the relatively long assay time (although highly variable depending on the technique used). Moreover, proteomic biomarkers for pain have not been sufficiently validated yet to incorporate such markers in pain assessment in the NICU.

### 3.4 Metabolomics

Metabolomics refers to the identification and quantification of small molecules (≤1,000 Da) that can be both substrates and products of cellular processes. The levels of these molecules are quickly affected by environmental factors such as diet, drugs or toxins, or the general state and health of a patient. The turnover, i.e., the production and half-life, of these molecules is usually shorter than in other omics, such as proteomics (134). Thus, measuring the metabolome can give a snapshot of a patient's current physical state. Measurement techniques for metabolomics include nuclear magnetic resonance (NMR), Fourier transform–mass spectrometry (FT-MS), gas chromatography–mass spectrometry (GC-MS), capillary electrophoresis–mass spectrometry (CE-MS), and liquid chromatography–mass spectrometry (LC-MS) (135).

Molecules that can be measured range from small hormones, such as epinephrine or norepinephrine, to stress metabolites such as cortisol, but also central energy metabolites or oxidative stress metabolites (136). Increases in glucose, lactate, pyruvate, free fatty acids, glycerol, and ketone bodies have been detected in preterm and term born neonates undergoing surgery (3, 85, 110, 112, 113). Analgesic therapy has been shown to reduce this rise in glucose and gluconeogenic substrates (3). In addition, analgesic therapy reduces the increase in urinary 3-MH/Cr ratio in the days following surgery (3), a measure of protein catabolism.

Further, responses to general stressors such as pain from repeating invasive procedures or chronic painful conditions can trigger metabolic cascades such as the catabolism of protein, fat, and carbohydrates. These may then cause conditions such as metabolic acidosis, hypoglycemia, hyperglycemia, and electrolyte imbalances, which can lead to increased morbidity and mortality in preterm or sick neonates (137).

Moreover, painful stimuli cause the release of endocannabinoids (e.g., anandamide, 2-arachidonoylglycerol). Endocannabinoids activate cannabinoid receptors, resulting in the inhibition of pain perception (138). It has been suggested that the analgesic effects of non-nutritive sucking are mediated by endocannabinoids (139). Further studies are needed to determine whether neonates’ endocannabinoid levels could serve as a marker for pain.

Although a quick bedside assay is available for glucose measurement, it does require collecting blood. In recent years, continuous blood glucose measurement devices have become available in neonates, but technical issues with the use of such devices in neonates still need to be solved (140). Other metabolites related to pain require blood collection as well, and have longer measurement times, making them less suitable for bedside pain assessment.

### 3.5 Oxidative stress

Acute pain in neonates has been related to oxidative stress (141–143). Oxidative stress is a state of imbalance in which the number of reactive oxygen species (ROS) exceeds the number of antioxidants (144). Although a moderate degree of oxidative stress is physiological (i.e., oxidative eustress), excessive oxidative stress (i.e., oxidative distress) can cause cell damage by for instance lipid peroxidation and glycoxidation reactions. Markers of oxidative stress include advanced oxidation protein products (AOPP), total hydroperoxides (TH), and malondialdehyde (MDA) (145).

In term born neonates undergoing painful procedures, an increase in blood levels of AOPP and TH has been observed which is proportional to the pain level (141, 142). In preterm neonates, elevated MDA levels have been detected following painful procedures (143). Oxidative stress following painful procedures may be attributed to reductions in oxygen saturation and increases in heart rate, resulting in enhanced energy expenditure and oxygen consumption (146). This is supported by the finding that reductions in oxygen saturation and increases in heart rate significantly correlated with increases in MDA levels (143). Other mechanisms that might contribute to oxidative stress in response to pain include tissue injury, inflammation, and cytokine production.

Similarly to most potential hormonal, proteomic, and metabolic biomarkers for pain, oxidative stress markers are measured in blood and therefore not suitable for bedside use in the NICU. Furthermore, studies evaluating the validity of oxidative stress markers for pain assessment in neonates are scarce.

## 4 Safety-biomarkers

Commonly used analgesics and sedatives in the NICU are acetaminophen, morphine, fentanyl, sufentanil, and midazolam (1). These analgesics and sedatives may cause adverse effects. Similarly to pain, these adverse effects may be monitored with so-called safety biomarkers including autonomic, proteomic, and metabolomic markers.

Potential adverse effects of opioids in preterm neonates include respiratory depression, bradycardia, hypotension, constipation, urinary retention, chest wall rigidity, and seizures (147, 148). These adverse effects can be detected by assessing vital signs, clinical symptoms, and EEG. In addition, concerns have been raised regarding the long-term neurodevelopmental effects of neonatal exposure to opioids. However, the devastating effects of opioid exposure identified in rodent studies have not been consistent in human studies (149, 150). Neurodevelopmental effects of opioids may be assessed with neuroimaging.

Midazolam use in preterm neonates has been related to bradycardia, hypotension, and myoclonus (151). These adverse effects can be monitored through physiological and clinical data, similarly to the adverse effects associated with opioid use.

While acetaminophen is generally well tolerated, it may cause hepatotoxicity (152), although these risks are low in newborns. Markers of hepatotoxicity include hepatic enzymes, bilirubin, and coagulation factor levels. A disadvantage of these widely used markers is that they follow rather than predict liver injury. Furthermore, they are not specific to acetaminophen-induced hepatotoxicity. Serum biomarkers based on the mechanism of acetaminophen-induced hepatotoxicity include acetaminophen protein adducts and serum acylcarnitines (153). Acetaminophen protein adducts are formed when high acetaminophen exposure depletes hepatic glutathione, which is responsible for detoxifying N-acetyl-p-benzoquinone imine (NAPQI), a toxic reactive acetaminophen metabolite (154, 155). Alterations in acylcarnitine levels are the result of disruptions in the fatty-acid beta-oxidation pathway due to mitochondrial dysfunction (156).

In addition to monitoring the presence of adverse effects, a more proactive approach may be employed by estimating serum concentrations of the administered analgesics, to ensure these are within the target range. This can be achieved using pharmacokinetic models, which predict serum concentrations of analgesics in neonates based on medication and clinical data (157–159).

## 5 Is there an ideal biomarker for assessing pain and pain relief?

**Figure 3:**
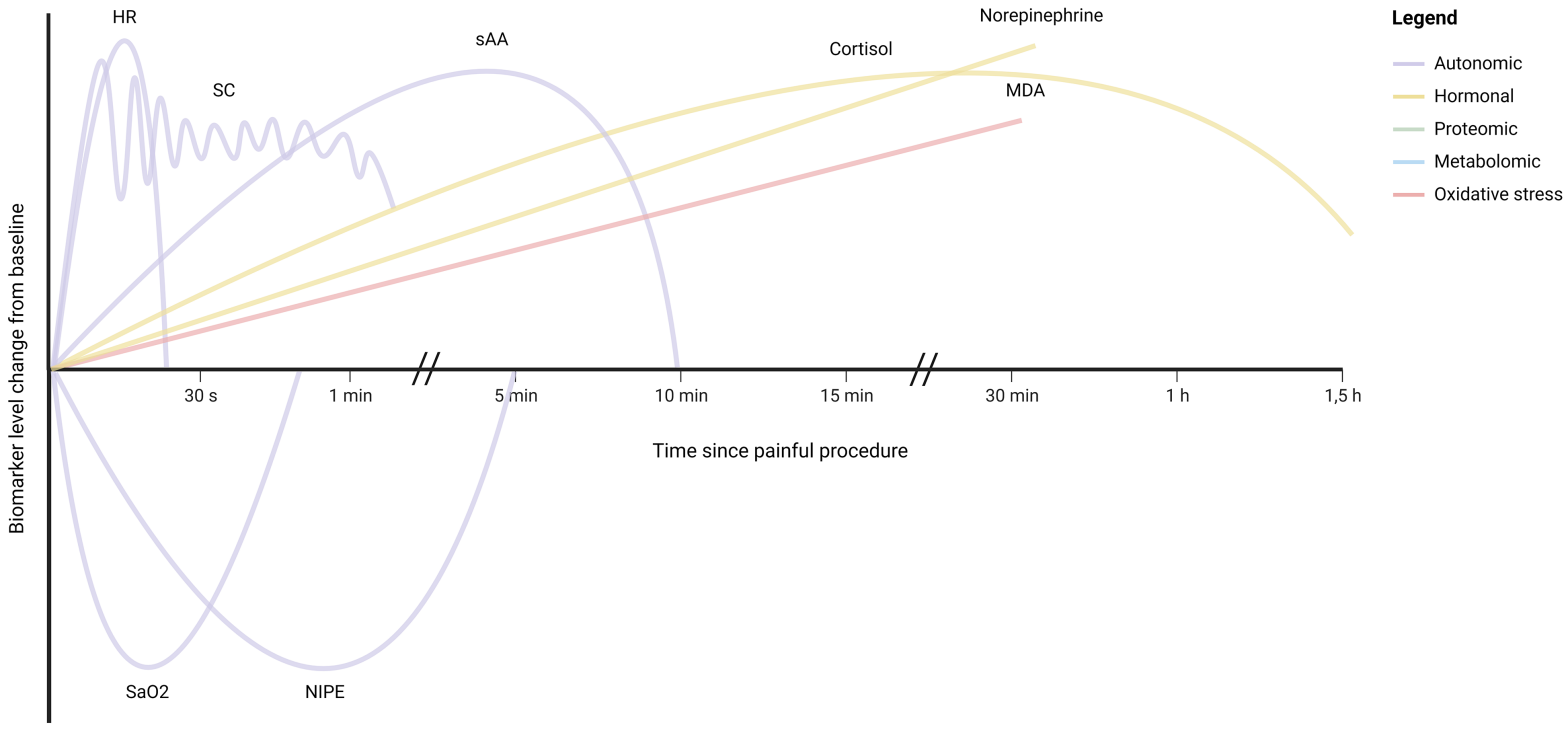
Illustration of the kinetics of monitoring biomarkers for neonatal pain, as estimated based on the available evidence from clinical studies. For illustrative purposes, the biomarker responses have been shown on the same y-axis scale, despite differences in unit and percentage change. HR, heart rate; SC, skin conductance; saO2, oxygen saturation; NIPE, newborn infant parasympathetic evaluation; sAA, salivary amylase; MDA, malondialdehyde. For biomarkers not represented in this illustration, no supporting studies were identified.

Figure 3 illustrates the kinetics of potential biomarkers for neonatal pain, for as far as they are currently known. Figure 2 illustrates the extent to which the described biomarkers possess the key properties for monitoring biomarkers for pain described in Section 2, namely validity, non-invasiveness, timeliness, and amount of available evidence, with the validity and amount of available evidence stratified by type of pain. As can be concluded from these two figures, the autonomic biomarkers quickly respond to pain and generally score high on availability, as these can be monitored continuously, but tend to score lower in terms of validity. Hormonal biomarkers exhibit the opposite pattern. Compared with procedural pain, fewer biomarkers have been evaluated for prolonged pain.

For evaluating the effectiveness of pain treatment with opioids, the autonomic biomarkers respiratory rate, heart rate, and blood pressure are likely less suitable, as opioids can also exert direct cardiorespiratory effects by activating µ-opioid receptors in areas involved in cardiovascular control and respiratory function (57, 160). Although the same receptors are responsible for the analgesic and respiratory effects of opioids, different pathways are involved, with the G-protein pathway being related to analgesia and the β-arresting pathway to adverse effects (161, 162). However, in the clinical setting, it cannot be distinguished whether a reduction in for instance respiratory rate is due to the analgesic effects of opioids or their impact on respiratory function.

For bedside pain assessment in the NICU, biomarkers that are invasive or not timely available are unsuitable (Figure 2). However, these biomarkers might be valuable for research purposes, especially if residual blood can be used to avoid additional blood collections. For both clinical and research purposes, validation and establishment of cutoff values for pain biomarkers are essential.

In addition to the properties shown in Figure 2, which determine biomarkers’ suitability for monitoring pain in theory, their practical application also depends on their availability at the NICU and their costs. Monitoring of physiological parameters is generally available at all NICUs, whilst more advanced monitoring (e.g., NIPE) and lab assays (e.g., omics) may not be readily available, especially in the resource-limited setting.

## 6 Discussion

Biomarkers for neonatal pain serve two purposes: pain assessment and evaluation of the effectiveness of pain treatment in the NICU. Currently, observational pain scores are employed as standard of care, which provide intermittent, mainly subjective assessments and may in certain situations be less valid (e.g., necrotizing enterocolitis, sedation). Integrating biomarkers may compensate for these limitations of current pain assessment.

Various biomarkers for pain in neonates have been studied, each with its advantages and disadvantages, but none of these single biomarkers seem to possess all the desired qualities for use in clinical care at the NICU, namely high validity, non-invasive collection, continuous availability, and robust support from literature (Figure 2). Therefore, a multimodal approach is likely needed, combining behavioral pain scales with different biomarkers. Autonomic biomarkers that respond rapidly to pain and can be continuously monitored might be used as a trigger for performing an observational pain score, thereby improving accuracy. Alternatively, advancements in artificial intelligence (AI) have facilitated the development of automatic tools for continuous multimodal pain assessment based on for instance movements and cry by video-analyses (163–165). A preliminary evaluation of one such tool by Zamzmi et al, which utilizes video (facial expression and body movements), audio, and vital signs for pain assessment, demonstrated a 95% accuracy, surpassing the accuracy of any of its individual components (166). Furthermore, rapid and accurate analysis of pain related changes in proteins and/or metabolites is now possible due to recent advancements in high resolution mass-spectrometry and neuro-imaging techniques. In the future, AI systems integrating video, audio, and autonomic biomarkers may enable automatic early pain detection, or even prediction (167).

For research purposes, continuous availability of a biomarker may not be required, but accuracy is crucial to obtain valid results. Hormonal, proteomic, metabolomic, or oxidative stress biomarkers may serve as endpoints of studies into the efficacy of analgesic therapy in neonates, which are highly needed. However, as highlighted by Figure 3, the kinetics of many of these biomarkers are yet to be unraveled in (preterm) neonates. Knowledge of the timeframe during which a pain biomarker can be detected is essential for its application in pain assessment. Previous studies evaluating biomarkers for neonatal pain often included only two time points, one before and one after a painful event, which limits the ability to evaluate the biomarker's time course. Future studies are needed to elucidate these kinetic profiles, in order to effectively incorporate biomarkers in pain assessment. Furthermore, for many biomarkers no measures for diagnostic performance are available, as existing studies only compared biomarker levels before and after a painful procedure in neonates, without relating them to pain scores. To establish the diagnostic performance of these biomarkers, further research is necessary, both for individual biomarkers and for combinations of biomarkers (multimodal approach). This is especially the case for prolonged pain, which has been studied far less than procedural pain (Figure 2). The validity of biomarkers may differ for the various types of pain (168). Further studies are needed to determine the validity of biomarkers for different types of pain in neonates, including for instance visceral pain related to necrotizing enterocolitis.

The NICU population is highly heterogeneous, with patients’ gestational ages ranging from extremely preterm to term born, their postnatal ages from the immediate postnatal period to several months after birth, and a myriad of medical conditions that may affect them including infections and neurological disorders. Therefore, multicenter collaborations are required to obtain large, high quality datasets that can enhance our understanding of the physiological and pathophysiological changes of biomarkers. Furthermore, the heterogeneous character of the NICU population underlines the need for multimodal pain assessment, as manifestations of pain likely vary between neonates. An aspect still underestimated and often neglected in research on biomarkers for pain in the NICU is the role of sex. Hence, it is highly recommended that future research on biomarkers for neonatal pain considers potential modifying effects of sex.

Many potential biomarkers for pain are based on the activation of the sympathetic nervous system or the HPA axis (Figure 1). In neonates admitted to the NICU, these systems may be activated by various physical (e.g., noises), psychosocial (e.g., separation from parents), and clinical stressors (e.g., NICU treatment), not exclusively pain (169). Pain has been defined as “an unpleasant sensory and emotional experience associated with, or resembling that associated with, actual or potential tissue damage” (170). Before incorporating biomarkers into (multimodal) pain assessment in the NICU, it is essential to examine their ability to distinguish pain and stress. Nonetheless, both pain and stress in neonates are important to detect to enable treatment.

In conclusion, integrating multiple pain biomarkers in pain management strategies in the NICU could be highly beneficial, but certain research gaps need to be addressed. Further studies are needed to establish the optimal approach for incorporating biomarkers in pain assessment in the NICU, with the ultimate goal of enabling valid, non-invasive, continuous bedside pain assessments, and thereby minimizing pain exposure in the NICU and optimizing neonatal outcomes.
